# A randomized controlled trial of motor imagery combined with structured progressive circuit class therapy on gait in stroke survivors

**DOI:** 10.1038/s41598-020-63914-8

**Published:** 2020-04-24

**Authors:** Sunee Bovonsunthonchai, Nilar Aung, Vimonwan Hiengkaew, Jarugool Tretriluxana

**Affiliations:** 1 Faculty of Physical Therapy, Mahidol University, Nakhon Pathom, Thailand; 2 Department of Physiotherapy, University of Medical Technology, Mandalay, Myanmar

**Keywords:** Rehabilitation, Stroke

## Abstract

Structured Progressive Circuit Class Therapy (SPCCT) was developed based on task-oriented therapy, providing benefits to patients’ motivation and motor function. Training with Motor Imagery (MI) alone can improve gait performance in stroke survivors, but a greater effect may be observed when combined with SPCCT. Health education (HE) is a basic component of stroke rehabilitation and can reduce depression and emotional distress. Thus, this study aimed to investigate the effect of MI with SPCCT against HE with SPCCT on gait in stroke survivors. Two hundred and ninety stroke survivors from 3 hospitals in Yangon, Myanmar enrolled in the study. Of these, 40 stroke survivors who passed the selection criteria were randomized into an experimental (n = 20) or control (n = 20) group. The experimental group received MI training whereas the control group received HE for 25 minutes prior to having the same 65 minutes SPCCT program, with both groups receiving training 3 times a week over 4 weeks. Temporo-spatial gait variables and lower limb muscle strength of the affected side were assessed at baseline, 2 weeks, and 4 weeks after intervention. After 4 weeks of training, the experimental group showed greater improvement than the control group in all temporospatial gait variables, except for the unaffected step length and step time symmetry which showed no difference. In addition, greater improvements of the affected hip flexor and knee extensor muscle strength were found in the experimental group. In conclusion, a combination of MI with SPCCT provided a greater therapeutic effect on gait and lower limb muscle strengths in stroke survivors.

## Introduction

Stroke is one of the top causes of long-term disability and mortality in many countries throughout the world^1,2^, with a high potential of this population increasing further due to the ageing population^3^. According to the disability-adjusted life years, stroke disease stands in fourth place among the disease burden. In 2005, there were 5.7 million deaths globally and 87% of them came from developing countries^4^.

Gait is one of the most important functions after stroke^5^. Stroke survivors usually exhibit gait alterations with longer stride time and lower gait speed and cadence when compared to aged matched healthy individuals^6^. Gait asymmetry is shown as one of the common characteristics in stroke survivors. It has been reported that 33.3% and 55.5% of ambulating stroke survivors had significant asymmetries in the temporal and spatial variables of gait^7^. Asymmetry of gait is clinically important and has been related to increases in energy expenditure, reduced balance control, and risk of unaffected limb injury^8^. The most important factor attributing to gait asymmetry is the reduction in muscle strength in the affected side. Previous studies exploring the relationship between lower limb muscle strength and walking ability, found significant associations in all muscle groups, especially in the hip flexors and ankle plantar flexors which showed the largest contribution to gait speed^9^. A review article reported that muscle weakness was one of the causation factors of falls, it is therefore considered to be the primary objective of promoting mobility ability in stroke survivors^10^.

Task-oriented training is one of several training techniques that has been used to improve motor function in stroke survivors^11–14^. This technique has been reported to improve functional tasks, allowing the patients to participate actively, and allows easy progression in the training levels and task adaptability^15^. The Structure Progressive Circuit Class Training (SPCCT) was developed based on the task-oriented training concept. The key components of this method are to provide group therapy with a minimum of 2 participants under 1 therapist supervisor and encouraging repeated practice exercises with continual progression^16^. This has advantages over other techniques and has been shown to increase therapy dosage and reduce treatment costs. This treatment technique may be suitable for a large number of patients, however, a limited number of therapists implement these techniques within the clinical setting.

Motor imagery (MI) is a cognitive function paradigm that involves the mental imitation of the movement without actual execution. MI has been used as part of training programs for a number of clinical conditions to improve motor ability, and has been shown to produce similar brain activity to real movement actions^17,18^. Imagination and motor planning are key parts of the brain’s capability to perform movement effectively. The purpose of MI training is to improve learning ability by repetitive practice of particular tasks^19^. Although studies support the practice with MI alone to improve lower limb function^20,21^, better results have been reported when MI was combined with physical training^22–24^. However, the previous studies have been conducted on upper limb function and only a few studies have reported its use in the lower limb^14,25^.

For a conservative treatment, health education (HE) is one of the crucial elements in stroke management. Stroke awareness is administered in the context of the national stroke policies in countries worldwide^26,27^. This provides knowledge about the disease and other necessary information for the patients and caregivers, and helps to inform patients how to take care of themselves as well as to prevent recurrence. This present study aimed to investigate the effect of the combined techniques of MI and SPCCT on gait and lower limb muscle strength on the affected side in stroke survivors. We hypothesized that the intervention of MI with SPCCT would show greater improvements when compared to HE with SPCCT.

## Methods

### Study design

This is a randomized double-blind balanced parallel-group (1:1) study.

### Participants

The study was conducted from January to May 2018 at the Physical Medicine and Rehabilitation department, North Okkalapa General Hospital, East General Hospital, and National Rehabilitation Hospital, Yangon, Myanmar. There were 290 patients with stroke screened for eligibility, 246 were excluded as they did not meet the selection criteria and 4 declined to participate. Thus, 40 patients were recruited to this study.

Inclusion criteria were (1) First event of stroke and unilateral involvement of the body, (2) Age 18 to 75 years, (3) Poststroke duration 3 to12 months, (4) Patients with middle cerebral artery lesion, (5) Able to walk a minimum of 10 meters with or without using assistive devices, (6) Functional Ambulation Category (FAC) ≥ 3, (7) Good cognition assessed using the Mini-Mental State Examination (MMSE) ≥ 24, (8) National Institutes of Health Stroke Scale (NIHSS) < 14, and (9) Good MI ability assessed by the Kinesthetic and Visual Imagery Questionnaire (KVIQ-10) ≥ 3. Participants were excluded if they presented with (1) Unstable cardiopulmonary problems (resting heart rate >120 bpm, resting systolic blood pressure >180 mmHg and resting diastolic blood pressure >100 mmHg), (2) Other neurological conditions such as Parkinson’s disease, Alzheimer’s disease, or epilepsy, (3) Orthopedic and rheumatologic disorders with weight-bearing pain, (4) Unable to communicate or unable to follow commands, (5) Serious cardiac conditions such as hospitalization for heart disease within 3 months, active angina, serious cardiac arrhythmias, hypertrophic cardiomyopathy, severe aortic stenosis, (6) Unilateral spatial neglect, (7) Ataxic movement, and (8) Under medication with muscle relaxing effects.

**Figure 1:**
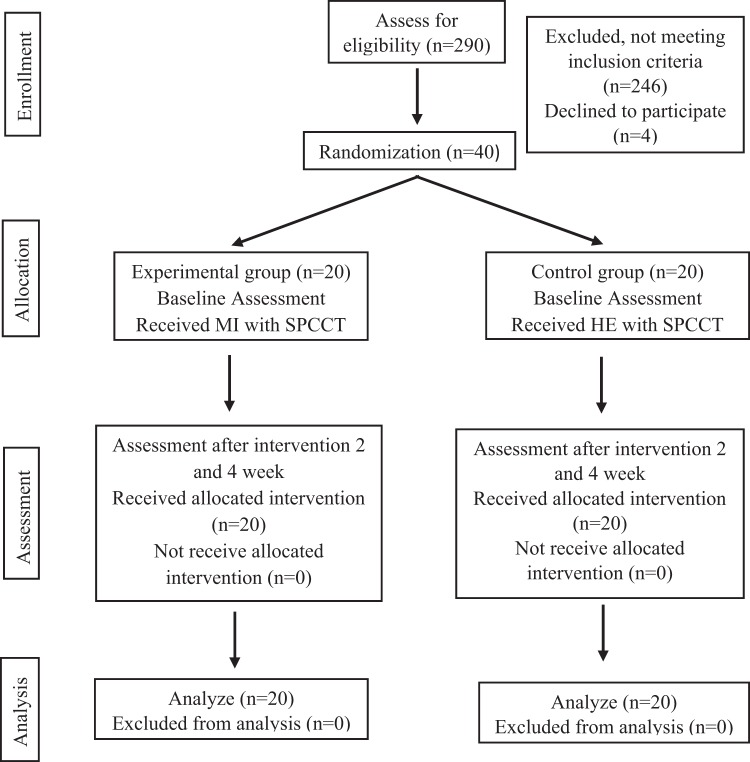
Flow chart diagram of the study.

The research was performed in accordance with the Declaration of Helsinki and the research protocol was approved by Mahidol University Central Institutional Review Board (MU-CIRB) (COA number: MU-CIRB 2017/178.1010). Participants were informed about the study and signed the consent form prior to participating in the study. After the baseline assessment, they were randomly allocated into the experimental or the control groups using a stratified random allocation technique. A flow chart diagram of the study is presented in Fig. 1.

### Procedure

#### Randomization and blinding

In this study, the allocation of the participants was performed by a computer-generated list of random numbers. In order to maintain recruitment balance between groups throughout the trial, a permuted block randomization process was used. Four blocks of five pairs using a random sequence allocation of participants was generated.

The sequence generation and allocation concealment were created by the researcher. In this study, sequentially numbered, opaque sealed envelopes (SNOSE) were provided to ensure adequate concealment of the allocation sequence^28^. The allocation envelopes were prepared by using aluminium foil and carbon paper. The aluminium foil was used to ensure that the allocation sheet inside could not be read when held against a light. The carbon paper was used to prevent violations of allocation concealment. Both the assessor and the participants were blinded to the study allocation.

#### Interventions

Both groups received the same protocol of SPCCT. The experimental group had 25 minutes of MI training preceded by 65 minutes of SPCCT, whereas the control group had 25 minutes of HE followed by 65 minutes of SPCCT, with both groups receiving the same overall intervention time of 90 minutes. Both groups practised 3 times a week for 4 weeks. Training schedules for the experimental and the control groups are presented in Table 1. Details of MI and SPCCT training programs are shown in Table 2.

**Table 1:** Training schedules for the experimental and the control groups.

Experimental group	Duration (min)	Control group	Duration (min)
**Motor imagery (MI):**		**Health Education (HE):**	
Phase 1: Relaxation	3	Explanation	12
Phase 2: MI practise (Visual) 4 tasks	8.5	Discussion	13
Rest	2		
Phase 3: MI practise (Kinesthetic) 4 tasks			
Phase 4: Refocusing	3		
Total time for MI	**25**	Total time for HE	**25**
**Structured Progressive Circuit Class Therapy (SPCCT):**		**Structured Progressive Circuit Class Therapy (SPCCT):**	
Warm up	3	Warm up	3
SPCCT practise 7 tasks (4 min practise, 4 min rest, and 1 min transfer in a task)	62	SPCCT practise 7 tasks (4 min practise, 4 min rest, and 1 min transfer in a task)	62
Total time for SPCCT	**65**	Total time for SPCCT	**65**
**Overall**	**90**	**Overall**	**90**

**Table 2:** Training tasks in the Motor Imagery (MI) and the Structured Progressive Circuit Class Training (SPCCT).

Tasks	Duration (min)
**MI**	
1. Stepping forward-backwards onto a block	2
2. Standing up from a chair, walking a short distance (3 m), and returning to the chair	2
3. Symmetrical walking (8 m)	2
4. Walking at a fast speed (8 m)	2
**SPCCT**	
Warm up	3
1. Stepping forward-backwards onto a block	9 (4 min practise, 4 min rest, 1 min transfer)
2. Stepping sideways onto a block	9
3. Heel lifts in standing to strengthen affected plantar-flexor muscles	9
4. Standing with a decreased base and reach for an object	9
5. Standing up from a chair, walking a short distance, and returning to the chair	9
6. Symmetrical walking	9
7. Walking at a fast speed	9

The training was conducted by the researcher in a silent room at the training centre. There were 4 phases of the MI training program. Before starting the MI practise participants were familiarized with the tasks. MI practise was performed in a sitting position with eyes closed the 1^st^ person perspective. For visual imagery, to visualize their movements being performed from inside of their body, as if they are looking at their own eyes while performing the movements. For kinaesthetic imagery, to perceive their body sensations of the movements without any movement. During the MI training, the participants were asked to keep track of the number of repetitions imagined with their fingers. Then, the tasks were progressed by increasing the speed, the rhythm of which was controlled with a metronome. The practise engagement was monitored using a heart rate monitor with oximeter^29,30^ and observed from their finger movements when counting the number of repetitions. After each practise, the number of practise performed were entered into a logbook and used as feedback for the next training session.

The twenty-five minutes of HE for the control group was conducted using pamphlets. The education program involved 12 minutes of explanation of health and 13 minutes discussion on the basis of patient’s problems. There were 6 topics of HE, composed of alteration after stroke, complications, emotional change, how to live at home, high blood pressure and stroke, and preventing recurrent stroke. One HE topic was provided for 2 treatment sessions and the 6 topics were delivered over the 4 weeks of the training program.

Physical therapists with 5 years of experience in the care of neurological conditions conducted the same SPCCT to both intervention groups. The SPCCT program involved 7 different workstations. The training for each participant was provided according to their condition regarding the levels of strength and mobility function status. The training of SPCCT was serial in order and distributed pattern and the participants performed the training in pairs. The patient who was in the rest phase observed while the other patient was in the training phase. To prevent the mental and physical fatiguability during the training protocol of MI and SPPCT, rest periods were provided. In addition, activities of participants were recorded in the logbook and used as the feedback for the next session. Training progression of the SPCCT at the next visit was carried out based on individual ability but controlled by practised duration. When the patients were able to perform the tasks easier, assessed using the score from self-confidence and physical therapist observations, progression was set at an increase in speed of 5–10% from the previous session.

### Outcome measures

All outcome measures were assessed by an experience physical therapist. The assessments were administered at baseline, 2 weeks, and 4 weeks after intervention.

#### Temporo-spatial gait variables

Temporo-spatial gait variables were measured using a 2D motion analysis system which has been show to be valid and reliable by a previous study on 20 stroke survivors^31^. The validity of the system was evaluated by comparing with a standard gait system (Force Distribution Measurement, FDM), with strong correlations (r_p_ = 0.94–0.99, p < 0.001) for all gait variables between the 2D motion analysis system and the FDM. In addition, this showed an excellent intra-rater reliability for all gait variables (ICC_3,1_ = 0.91–0.99, p < 0.001).

The FDM consisted of an electronic walkway 307 cm long, 60.5 cm wide, and 2.1 cm deep that was connected to a personal computer. For the calibrated reference, two paper tapes measures of 55 cm in length were attached in the centre of the walkway, aligned to each of the left and the right foot progression. A tripod fixed with a video camera was placed 3 meters away from the walkway and focused on the middle part to record around 3 gait cycles of sagittal plane motion. The Kinovea software version 0.8.15.0 was used to measure step length and step time of both limbs. The measurement involved 3 trials of walking at the preferred gait speed along the 8 meters walkway. One physiotherapist followed the participants to prevent tripping or falling during the gait testing. However, no trips or falls were reported during data collection.

A gait cycle in the middle part of the walkway was selected for data processing and the averaged values from 3 trials were used for the comparisons. The initial contacts of each limb were marked on the video using the Kinovea software, and step length and step time of the left and right sides were measured.

The distance from the point of initial contact of one limb to the contralateral limb was measured for step length. The actual length of each side was calculated in reference to the two calibrated rulers. The step time of the limbs was calculated from the time taken from the point of initial contact of one limb to the point of initial contact of the other limb. Stride length and stride time were calculated from the sum of left and right step lengths and left and right step times. Gait speed was calculated from stride length divided by stride time. Cadence was calculated from gait speed multiplied by 120 and divided by stride length.

### Symmetry index (SI)

The SI of step length and step time was calculated using the following formula;

$${\rm{SI}}=|\frac{({{\rm{X}}}_{{\rm{unaffected}}}-{{\rm{X}}}_{{\rm{affected}}})}{0.5({{\rm{X}}}_{{\rm{unaffected}}}+{{\rm{X}}}_{{\rm{affected}}})}\times 100|$$

where X is the step length/step time

### Muscle strength of the lower limb

To measure the maximum voluntary isometric contraction of the affected hip flexor and extensor, knee flexor and extensor, ankle dorsiflexor and plantarflexor, a hand-held dynamometer (The Lafayette Manual Muscle Tester, 16200858, USA) was used. This has been reported to be a reliable and valid instrument and has been used in the assessment of stroke survivors^32^. The protocol of lower extremity muscle strength testing used in this study was adapted from a previous study performed in the elderly^33^. The positioning for the assessment for all muscles was set in a side-lying position on the unaffected side to eliminate gravity effect and provide stabilization for the patients. Patients were asked to exert the maximum force against the dynamometer. One practice trial for each muscle group was allowed before testing to ensure that the patients fully understood the assessment task. The details of positioning and dynamometer placement for each muscle group testing are presented in Table 3.

**Table 3:** The protocol of muscle strength testing for the affected lower limb muscle.

Muscle group	Position	Dynamometer placement
Hip flexor	Unaffected limb is in a neutral position and affected limb is on the mat table, hip flexed to 45°, knee flexed to 60°	Mid-point on the anterior surface of the distal part of the femur (10 cm above the patella)
Hip extensor	Hip and knee of the affected limb extended on the mat table	Mid-point on the posterior surface of the distal part of the femur (same level of hip flexor)
Knee flexor	Affected hip flexed to 45°, knee flexed to 60° on the mat table	Posterior side of the distal part of the tibia (10 cm above the lateral malleolus)
Knee extensor	Affected hip flexed to 45°, knee flexed to 60° on the mat table	Anterior side of the distal part of the tibia (10 cm above the lateral malleolus)
Ankle dorsiflexor	Affected hip and knee extended, ankle neutral position on the mat table	Mid of the dorsal surface of the foot, just proximal to the metatarsal phalangeal
Ankle plantarflexor	Affected hip and knee extended, ankle neutral position on the mat table	Mid of the plantar surface of the foot, just proximal to the metatarsal phalangeal

### Sample size calculation

The sample size was estimated by the G*Power 3.1 software using pilot data from a sample of 10 stroke survivors. To estimate the sample size for a between-group comparison, the analysis was performed based on stride length. Effect size (f) was set at the medium level with 0.26, power of 80%, 2 tailed tests, and an alpha error probability set at 0.05. The total sample size was estimated to be 40. Thus, the number of participants in this study was sufficient.

### Statistical analysis

All analysis was performed using SPSS version 18.0. The study presented demographic and clinical characteristics of the participants by using the mean, standard deviation, and range for the continuous variables. Frequency and percentage were presented for the categorical variables. The Kolmogorov Smirnov Goodness of Fit test was used to test the data distribution. The significant difference was set at p < 0.05 for all comparisons.

For the normally distributed variables i.e. step length of the affected and the unaffected limbs, stride length, step time of the affected and the unaffected limbs, gait speed, cadence and affected muscle strength, a two-way mixed methods repeated measure ANOVA was performed to investigate differences between the factors of group and time. The main effect of group (treatment) and time (assessments), as well as the interaction effects of group by time were explored.

For the non-normally distributed variables i.e. SI of step length and step time, the Mann-Whitney U test (between-group) and the Friedman test in conjunction with the Wilcoxon Signed-Rank test were used to explore the within group differences between time points.

## Results

### Participants’ characteristics

Table 4 represents the baseline characteristics of the experimental and control groups. Forty stroke survivors participated in the study, 26 males and 14 females. There was no significant difference (p > 0.05) in all demographic data including age, height, body weight, sex or other baseline data such as time since stroke, Functional Ambulation Category (FAC), Mini-Mental State Exam (MMSE), National Institute of Health Stroke Scale (NIHSS), MI ability, type of stroke, side of involvement, and severity.

**Table 4:** Demographic characteristics of the experimental and the control groups (n = 40).

Characteristics	Experimental (n = 20)	Control (n = 20)	p-value
Mean age (years)	49.90 ± 11.59	55.55 ± 10.74	0.837^a^
Mean height (cm)	163.89 ± 6.55	162.03 ± 13.04	0.210^b^
Mean body weight (kg)	63.65 ± 6.63	63.35 ± 13.04	0.228^b^
Mean time since stroke (weeks)	25.00 ± 15.46	28.85 ± 12.94	0.350^b^
Functional Ambulation Category (FAC)			
3	8 (40%)	8 (40%)	1.000^b^
4	12 (60%)	12 (60%)	
Mini Mental State Exam (MMSE)	26.80 ± 1.11	26.85 ± 0.93	0.977^b^
National Institute of Health Stroke Scale (NIHSS)	3.45 ± 1.39	3.15 ± 1.31	0.553^b^
Motor Imagery ability (scores)	38.50 ± 4.62	37.00 ± 5.94	0.385^b^
Gender			
Male, n (%)	15 (75%)	11 (55%)	0.320c
Female, n (%)	5 (25%)	9 (45%)	
Type of stroke			
Ischemic, n (%)	12 (60%)	14 (70%)	0.741^c^
Hemorrhage, n (%)	8 (40%)	6 (30%)	
Side of involvement			
Right, n (%)	10 (50%)	12 (60%)	0.751^c^
Left, n (%)	10 (50%)	8 (40%)	
Severity			
Mild, n (%)	12 (60%)	14 (70%)	0.741^c^
Moderate, n (%)	8 (60%)	6 (30%)	

Significant difference tested by ^a^Independent sample t-test; ^b^Mann-Whitney U test; ^c^Fisher Exact test at p < 0.05.

### Comparisons of the temporo-spatial gait variables

Table 5 shows comparisons of the temporo-spatial gait variables between the experimental and control groups at each time point of assessment; baseline, 2 weeks, and 4 weeks after the intervention within each group.

**Table 5:** Comparisons of the spatiotemporal gait variables between the experimental and control groups at each time point of assessment and among baseline, 2 weeks, and 4 weeks after the intervention within each group.

Outcome measure	Group	Baseline	2 week after	4 week after	p-value ^b^	p-value ^c^	p-value ^d^
Step length (affected) (cm)	Experimental	37.11 ± 8.33	43.91 ± 8.44	49.76 ± 6.99	**<0.001**	**<0.001**	**<0.001**
	Control	35.47 ± 12.01	39.33 ± 10.95	42.07 ± 11.46	**0.031**	**<0.001**	**0.001**
	**p-value**^**a**^	0.602	0.146	**0.015**			
Step length (unaffected) (cm)	Experimental	34.19 ± 12.70	41.98 ± 9.98	47.78 ± 8.37	**<0.001**	**<0.001**	**<0.001**
	Control	34.47 ± 11.39	37.28 ± 11.03	41.45 ± 12.17	0.118	**<0.001**	**<0.001**
	**p-value**^**a**^	0.942	0.166	0.063			
Stride length (cm)	Experimental	71.71 ± 18.70	86.33 ± 16.89	97.34 ± 14.87	**<0.001**	**<0.001**	**<0.001**
	Control	69.94 ± 22.60	76.61 ± 21.39	83.07 ± 22.72	**0.030**	**<0.001**	**0.001**
	**p-value**^**a**^	0.788	0.119	**0.024**			
Step time (affected) (s)	Experimental	1.01 ± 0.29	0.89 ± 0.22	0.70 ± 0.13	**<0.001**	**<0.001**	**<0.001**
	Control	1.07 ± 0.56	1.02 ± 0.52	0.95 ± 0.49	**0.033**	**0.004**	**0.022**
	**p-value**^**a**^	0.647	0.324	**0.038**			
Step time (unaffected) (s)	Experimental	0.76 ± 0.24	0.66 ± 0.16	0.57 ± 0.12	**0.009**	**<0.001**	**<0.001**
	Control	0.89 ± 0.38	0.84 ± 0.34	0.77 ± 0.32	0.378	**0.007**	**<0.001**
	**p-value**^**a**^	0.195	**0.039**	**0.011**			
Gait speed, (m/s)	Experimental	0.44 ± 0.18	0.58 ± 0.20	0.78 ± 0.21	**<0.001**	**<0.001**	**<0.001**
	Control	0.43 ± 0.24	0.49 ± 0.24	0.58 ± 0.27	**<0.001**	**<0.001**	**<0.001**
	**p-value**^**a**^	0.888	0.236	**0.015**			
Cadence (Steps/min)	Experimental	72.77 ± 19.15	79.61 ± 15.3	94.13 ± 17.09	**0.002**	**<0.001**	**<0.001**
	Control	70.26 ± 25.64	73.85 ± 24.05	78.88 ± 26.35	0.176	**0.009**	**0.036**
	**p-value**^**a**^	0.728	0.371	**0.036**			

Parametric data: Two-way mixed repeated ANOVA, Pairwise comparison tested by the Bonferroni Post hoc test, Significant difference was tested at p < 0.05.

Between-group comparison; ^a^Comparison of the variables at baseline, 2 weeks after, and 4 weeks after intervention between the experimental and control groups.

Within-group comparison; ^b^Comparison of the variables in each group between baseline and 2 weeks after the intervention; ^c^ Comparison of the variables in each group between baseline and 4 weeks after the intervention; ^d^Comparison of the variables in each group between 2 weeks and 4 weeks after intervention.

There was no significant effect of group but a significant effect of time in step length on the affected limb [F (1, 38) = 2.485, p = 0.123], [F (2, 76) = 56.248, p < 0.001], step length of the unaffected limb [F (1, 38) = 1.158, p = 0.289], [F (2, 76) = 64.621, p < 0.001], stride length [F (1,38) = 2.076, p = 0.158], [F (2,76) = 72.317, p < 0.001], step time of the affected limb [F (1,38) = 1.347, p = 0.253], [F (2,76) = 61.173, p < 0.001], gait speed [F (1,38) = 1.893, p = 0.177], [F (2,76) = 192.792, p < 0.001] and cadence [F (1,38) = 1.403, p = 0.244], [F (2,76) = 47.974, p < 0.001]. There was significant effect of group and time in step time in the unaffected limb [F (1,38) = 4.171, p = 0.048], [F (2,76) = 29.488, p < 0.001].

A significant interaction effect of group by time was seen for step length in the affected limb [F (2, 76) = 5.540, p = 0.006], step length in the unaffected limb [F (2, 76) = 7.251, p = 0.001], stride length [F (2,76) = 7.686, p = 0.001], step time of the affected limb [F (2,76) = 11.085, p < 0.001], gait speed [F (2,76) = 28.625, p < 0.001] and cadence [F (2,76) = 9.064, p < 0.001] were found. There was no significant interaction effect between group by time for step time in the unaffected limb, [F (2,76) = 1.538, p = 0.221].

For the between group comparisons, a difference was found only in the step time of the unaffected side 2 weeks after intervention (p = 0.039). At 4 weeks after the intervention, there were significant differences (p < 0.05) in almost all temporo-spatial gait variables, except for the step length of the unaffected limb (p = 0.063).

For within-group comparisons, significant differences were found in all temporo-spatial variables (p < 0.001) between pairs of comparisons for both groups. Except for step length and step time of the unaffected side and cadence that showed no differences between baseline and 2 weeks after intervention in the control group (p > 0.05).

### Comparisons of the symmetry indices (SI) of step length and step time

Table 6 shows comparisons of the SI of the step length and step time between the experimental and control groups at each time point of assessment and among baseline, 2 weeks, and 4 weeks after the intervention within each group.

**Table 6:** Comparisons of the symmetry indexes (SI) of step length and step time between the experimental and control groups at each time point of assessment and among baseline, 2 weeks, and 4 weeks after the intervention within each group.

Outcome measure	Group	Baseline	2 week after	4 week after	p-value ^b^	p-value ^c^	p-value ^d^
SI Step length (%)	Experimental	12.30 (7.61–44.06)	6.35 (2.40–22.52)	0.44 (0.22–5.13)	**0.001**	**<0.001**	**<0.001**
	Control	12.64 (6.40–23.62)	5.53 (2.32–18.37)	8.19 (2.21–15.61)	**<0.001**	**0.004**	0.930
	**p-value**^**a**^	0.829	0.978	**0.004**			
SI Step time (%)	Experimental	29.00 (7.85–44.49)	26.11 (10.79–48.83)	19.19 (9.24–38.78)	0.601	0.156	0.117
	Control	27.49 (10.15–49.09)	28.06 (10.20–52.65)	26.35 (12.42–57.82)	0.970	0.765	0.940
	**p-value**^**a**^	0.749	0.735	0.433			

Nonparametric data: Wilcoxon Signed-Rank test, Mann-Whitney U test; Significant difference tested at p-value < 0.05;

Between-group comparison; ^a^Comparison of the variables at baseline, 2 weeks after and 4 weeks after intervention between the experimental and control groups;

Within-group comparison; ^b^Comparison of the variables between baseline and 2 weeks after the intervention; ^c^Comparison of the variables between baseline and 4 weeks after the intervention; ^d^Comparison of the variables between 2 weeks and 4 weeks after intervention.

For between-group comparisons, a significant difference was found only in the SI of step length at 4 weeks after the intervention. Whereas, the other time points of assessment and the SI of step time showed no differences.

For SI of step length, significant differences were found between all pairwise comparisons in the experimental group (p < 0.05). In addition, the control group showed differences between baseline and 2 weeks after intervention (p < 0.001) and between baseline and 4 weeks after intervention (p = 0.004), however, there were no differences for the SI of step time in both groups (p > 0.05).

### Comparisons of muscle strength on the affected side

Table 7 shows comparisons of the affected muscle strength between the experimental and control groups at each time point of assessment and among baseline, 2 weeks, and 4 weeks after the intervention within each group.

**Table 7:** Comparisons of the muscle strengths between the experimental and control groups at each time point of assessment and among baseline, 2 weeks, and 4 weeks after the intervention within each group.

Outcome measure	Group	Baseline	2 week after	4 week after	p-value^b^	p-value^c^	p-value^d^
Hip flexor (kg)	Experimental group	2.58 ± 0.44	3.07 ± 0.49	3.46 ± 0.66	**<0.001**	**<0.001**	**<0.001**
	Control group	2.38 ± 0.36	2.60 ± 0.30	2.89 ± 0.38	**0.015**	**0.001**	**<0.001**
	**p-value**^**a**^	0.113	**0.001**	**0.002**			
Hip extensor (kg)	Experimental group	2.54 ± 0.49	2.96 ± 0.64	3.38 ± 0.73	**<0.001**	**<0.001**	**<0.001**
	Control group	2.49 ± 0.45	2.71 ± 0.39	3.06 ± 0.46	**0.004**	**<0.001**	**<0.001**
	**p-value**^**a**^	0.789	0.136	0.107			
Knee flexor (kg)	Experimental group	2.09 ± 1.16	2.46 ± 1.24	2.83 ± 1.41	**<0.001**	**<0.001**	**<0.001**
	Control group	2.15 ± 0.84	2.35 ± 0.90	2.59 ± 1.00	**0.006**	**0.001**	**<0.001**
	**p-value**^**a**^	0.853	0.749	0.548			
Knee extensor (kg)	Experimental group	2.49 ± 0.41	3.14 ± 0.49	3.75 ± 0.71	**<0.001**	**<0.001**	**<0.001**
	Control group	2.31 ± 0.42	2.68 ± 0.50	3.19 ± 0.64	**0.001**	**<0.001**	**<0.001**
	**p-value**^**a**^	0.157	**0.006**	**0.014**			
Ankle dorsiflexor (kg)	Experimental group	1.54 ± 0.98	1.88 ± 1.04	2.27 ± 1.14	**0.004**	**<0.001**	**<0.001**
	Control group	1.91 ± 1.07	2.06 ± 1.12	2.28 ± 1.24	0.372	0.062	**<0.001**
	**p-value**^**a**^	0.261	0.602	0.979			
Ankle Plantarflexor (kg)	Experimental group	1.64 ± 0.96	2.03 ± 1.14	2.52 ± 1.26	**<0.001**	**<0.001**	**<0.001**
	Control group	1.8 ± 0.86	2.06 ± 0.88	2.37 ± 0.99	**0.003**	**<0.001**	**0.004**
	**p-value**^**a**^	0.570	0.939	0.668			

Parametric data: Two-way mixed repeated ANOVA, Pairwise comparison tested by Bonferroni Post hoc test, Significant difference was tested at p < 0.05.

Between-group comparison; Comparison of the variables at baseline, 2 weeks after and 4 weeks after intervention between the experimental and control groups;

Within-group comparison; ^b^Comparison of the variables between baseline and 2 weeks after the intervention; ^c^Comparison of the variables between baseline and 4 weeks after the intervention; ^d^Comparison of the variables between 2 weeks and 4 weeks after intervention.

There was no significant effect of group but a significant effect of time for strength of the hip flexor on the affected side [F (1,38) = 11.492, p = 0.202] [F (2,76) = 57.709, p < 0.001], hip extensor [F (1,38) = 1.685, p = 0.202], [F (2,76) = 78.561, p < 0.001], knee flexor [F (1,38) = 0.076, p = 0.785] [F (2,76) = 55.414, p < 0.001], ankle dorsiflexor [F (1, 38) = 0.309, p = 0.582], [F (2, 76) = 23.870, p < 0.001], ankle plantarflexor [F (1, 38) = 0.001, p = 0.970], [F (2, 76) = 48.020, p < 0.001]. For the knee extensor, significant effects of group [F (1,38) = 7.851, p = 0.008] and time [F (2,76) = 84.543, p < 0.001] were found.

The significant effect of group by time for the hip flexors [F (2,76) = 4.343, p < 0.016], hip extensor [F (2,76) = 3.383, p = 0.039], knee flexor [F (2, 76) = 3.459, p = 0.036], ankle dorsiflexor [F (2, 76) = 2.552, p = 0.085] were found. There was no significant effect of group by time for the knee extensors [F (2, 76) = 2.637, p = 0.078] and ankle plantarflexor [F (2, 76) = 2.343, p = 0.103].

There were significant differences in the affected side muscle strength between the experimental and control groups for the hip flexor at 2 weeks after the intervention (p = 0.001) and at 4 weeks after the intervention (p = 0.002) and for the knee extensor at 2 weeks after the intervention (p = 0.006) and at 4 weeks after the intervention (p = 0.014). In contrast, there were no differences (p > 0.05) in the other muscle strength measures between groups.

Significant differences (p < 0.05) were found in all pairwise comparison for all muscle strengths, except for the ankle dorsiflexor strength in the control group which showed differences between 2 weeks and 4 weeks after the intervention (p < 0.001).

## Discussion

In the present study, the experimental group demonstrated significant improvements in almost all spatiotemporal variables, except for the step length in the unaffected limb and step time symmetry, when compared to the control group 4 weeks after the intervention. These improvements probably resulted from the combined benefit of MI and SPCCT. Practising with similar tasks using the MI method prior to the real task training in SPCCT, helped the patients to improve movement ability, initiating with the awareness of their movement. This mental representation acted as the preparation phase before the actual movements were trained. In general, the ability to perform MI involves previous experiences of the specific movement or task and depends on the working memory which can affect motor representation internally. MI was provided with visual and kinesthetic imageries from the first-person perspective. This method may enhance motor performance by neural plasticity and brain activation in the motor-related areas and parietal lobe^18,34^. In addition, the program of SPCCT was structured with the principles of motor control and motor learning theories. This provided task-specific training involving a higher level of functional activities.

However, no difference in the step length on the unaffected limb was found, this may be due to a greater variability. This is supported by a previous study which compared stroke survivors and healthy individuals and showed a greater variability in step length especially on the unaffected limb in the stroke survivors^35^. Moreover, step length of the unaffected limb has been reported to be associated with ankle plantarflexor spasticity^36^ and the MI or SPCCT training protocol in the present study did not target a reduction in spasticity. Similarly, no difference in step time symmetry was found in this study. This may be due to a large difference between the affected and unaffected limbs at baseline. In addition, this may be related to the degree of ankle dorsiflexor and plantarflexor spasticity that resulted in deficits during weight acceptance and force generation during push-off, therefore contributing to the asymmetry in step time^36,37^.

The improvements of almost all gait variables found in this study may originate from the relationship between the gait variables, with improvements in one variable resulting in improvements in the other variables. For instance, an increase in step length bilaterally can improve stride length and overall gait speed. The greater improvement in step length seen in the experimental group may come from the visual imagery training, improving spatial accuracy due to the attention on the visuospatial aspects of the task. In our study, at 4 weeks after the intervention, improvements in gait speed showed an increase of 0.34 m/s in the experimental group and by 0.15 m/s in the control group. This improvement is above what might be considered as a clinically important change within the experimental group. When compared to the findings from previous studies that investigated the effect of MI, and various types of task-oriented training or circuit class training in stroke survivors, gait speed was only increased by around 0.14–0.17 m/s^14,20,38^. The improvement of gait speed seen in our study, showed almost twice the level of improvement seen in previous studies. Amongst the tasks practised in using MI and SPCCT were; sit-to-stand, reaching in sitting and standing, marching, walking, turning and transfers. Therefore, patients appeared to be able to learn the tasks through repetition using the MI paradigm followed by the real execution of the tasks. The improvement of gait speed may also be derived from the training program which emphasized a longer duration of weight-bearing on the affected side to improve strength, as well as the instruction used to exert a greater force during the push-off phase. Moreover, a metronome was also used to train step rhythm which was used within the progressive training of SPCCT.

Cadence or walking rate, which is calculated from the number of steps taken over one minute, and is widely used as an indicator of ambulatory status and the assessment of physical activity level^39^. A significant positive relationship was found between cadence and community reintegration in stroke survivors^40^. In addition, a cadence of more than 100 steps/min has been suggested to be the threshold value setting of moderate-intensity ambulatory activity in normal healthy adults^39^. After 4 weeks of training, we found an increase in cadence of 22 steps/min for the experimental group and 9 steps/min for the control group. The experimental group had a clear increase in cadence from 72 to 94 steps/min. While the previous studies reported an increase in cadence of 4.84 steps/min after MI with treadmill training^41^ and 19.13 steps/min after MI with walking-related tasks in task-oriented circuit class training^14^. The increase in cadence seen in this study may be as a result of the constituent parts of the training program that consisted of stepping and walking tasks which were combined with external cues using a metronome and marker cues on the floor.

For gait symmetry, we only found a significant improvement in step length symmetry in the experimental group at 4 weeks after the intervention. For gait symmetry training attaining both legs to step the same distance and time was quite difficult in this patient group. However, when considering the data at 4 weeks, there was a tendency towards an improvement of symmetry in both step length and step time for the experimental group. This is supported by Verma *et al*.^14^ who found no significant effect of MI and task-oriented circuit class training on step length symmetry, and offered the explanation that the training program was not primarily designed for improve gait symmetry. So, the specific training protocol is important regarding the targeted outcome. The present study provided stepping tasks bilaterally to improve step length on both sides, standing and reach task to enhance weight shift and pelvic stability, followed by symmetrical walking training which aimed to improve gait symmetry.

It is well established that skeletal muscle strength can be developed through morphological and neurological adaptations^42^. Previous studies support that improvements in muscle strength may be achieved through MI as the control of movement is associated with the activation of motor-related areas of the brain^38,42^ and enhanced cortical output signals^29^ during MI practice. In our study, there were significant improvements in muscle strengths in the affected hip flexor and knee extensor at 2 weeks and 4 weeks after the intervention in the experimental group when compared to the control group. Whereas, the other lower limb muscles showed no difference. In contrast, Kumar *et al*.^38^ investigated the effect of MI and task-oriented training in stroke survivors and a healthy control group. They found a significant improvement of almost all lower limb muscles on the affected side in the experimental group. This may be explained by the use of different protocols of MI training as well as training intensity and duration used in the different studies. The improvement of strength in these two muscles may be related to the improvement in gait speed, as it has been reported that the key muscles to determine gait speed are the hip flexors and knee extensors^37^. This result was consistent with Yang *et al*.^12^ who studied task-oriented progressive resistance strength training, and found that it could improve lower limb muscle strength, resulting in improvements in lower limb function^12^. The present study found comparable results with the previous MI with Task-specific training studies^14,38^ on gait performance. The tasks of MI in the present study were the same as those used in the SPCCT, therefore the similarity of the tasks may lead to a maximal transfer effect of the training^43^. The SPCCT of the present study was structured and progressed according to the individual condition of the patients.

This study may be limited by the retention effect of the training program which did not investigate the improvement of gait performance, but did consider the physical performance improvement without the effects on neuroplasticity. These results would suggest a benefit of a combined MI and SPCCT program which may be generalized to a similar stroke population group as tested in this study, although a larger sample size may highlight further benefits.

## Conclusion

The combined interventions of MI and SPCCT provided benefits with improvements in the spatiotemporal gait variables and affected muscle strength over the use of HE and SPCCT. We recommend physiotherapists to use MI combined with exercise than exercise alone in the management of stroke survivors.

## Data Availability

All data are freely available from the authors on request.
